# Helminths of Wied’s marmoset (*Callithrix kuhlii* (Coimbra-Filho, 1985) (Primates: Callitrichidae)) from the Atlantic Forest, Southern Bahia State, Brazil

**DOI:** 10.1590/S1984-29612024011

**Published:** 2024-02-23

**Authors:** Aléxia David Santos Soares, Márcio Borba da Silva, Ricardo Evangelista Fraga, Estevam Guilherme Lux Hoppe, Wilson Junior Oliveira, Alexandre Schiavetti

**Affiliations:** 1 Laboratório de Zoologia - Labzoo, Instituto Multidisciplinar em Saúde, Universidade Federal da Bahia - UFBA, Campus Anisio Teixeira, Vitória da Conquista, BA, Brasil; 2 Laboratório de Etnoconservação e Áreas Protegidas - LECAP, Universidade Estadual de Santa Cruz - UESC, Ilhéus, BA, Brasil; 3 Laboratório de Biologia celular e Biologia Molecular, Instituto Multidisciplinar em Saúde, Universidade Federal da Bahia - UFBA, Campus Anisio Teixeira, Vitória da Conquista, BA, Brasil; 4 Laboratório de Enfermidades Parasitárias - LabEPar, Universidade Estadual Paulista - UNESP, Jaboticabal, SP, Brasil

**Keywords:** Neotropical Primates, alpha taxonomy, morphology, Primatas neotropicais, taxonomia-alfa, morfologia

## Abstract

*Callithrix kuhlii* is present in forest mosaics, edge habitats, and abandoned fields in the Atlantic Forest. In Bahia and Minas Gerais. This study aimed to identify helminths from *C. kuhlii* and relate them to the clinical data, weights, and indices of the liver and gonads. Necropsies were performed on 13 adult marmosets that were run over on the BA-001 highway. A principal component analysis (PCA) was conducted to describe the relationships between the variables investigated. Fifty-one helminths were collected from 30.77% (4/13) of the marmosets analyzed. Helminths were classified based on their morphological and morphometric characteristics. *Primasubulura jacchi* (Marcel, 1857), *Platynosomum illiciens* (Dougherty, 1946), and *Prosthenorchis confusus* (Dougherty, 1946) were the species identified, with prevalence rates of 7.69%, 7.69%, and 15.38%, respectively. In addition, this is a new host record of *P. confusus*. The two main axes of the PCA explained a high variability (PCA=67.7%), indicating reduced weight and indices of the organs of parasitized animals. This study expands the knowledge on parasites of *C. kuhlii* and its vulnerability to parasites, contributing to constructing an epidemiological profile of environmental health.

## Resumo

*Callithrix kuhlli* é encontrado em mosaicos florestais, habitats de borda e campos abandonados da Mata Atlântica. Na Bahia e Minas Gerais. O objetivo do trabalho foi identificar helmintos recuperados de *C. kuhlli* e relacioná-los aos dados clínicos, pesos e índices provenientes do fígado e das gônadas. Foram realizadas necropsias em 13 saguis adultos atropelados na rodovia BA-001 e realizada uma análise de componentes principais (PCA), para descrever as relações entre as variáveis. Recuperou-se 51 helmintos em 30,77% (4/13) dos saguis analisados. A classificação dos helmintos ocorreu por meio das características morfológicas e morfométricas, sendo *Primasubulura jacchi* (Marcel, 1857), *Platynosomum illiciens* (Dougherty, 1946) e *Prosthenorchis confusus* (Dougherty, 1946) as espécies identificadas, com prevalências de 7,69%; 7,69% e 15,38% respectivamente. Ainda, *P. confusus* apresenta um novo registro de hospedeiro. Já os dois eixos principais da PCA explicaram alta variabilidade (PCA=67,7%), verificando nos animais parasitados uma redução do peso e índices provenientes dos órgãos. Este trabalho amplia o conhecimento sobre parasitos do *C. kuhlli* e o grau de vulnerabilidade aos parasitas, contribuindo para uma possível construção de perfil epidemiológico da saúde ambiental.

## Introduction

Marmosets from the genus *Callithrix* spp. are omnivorous and greatly adapt to secondary and anthropized environments (Coimbra-Filho & Mittermeier, 1976; Rylands, 1989). This primate is present in forest mosaics, including cocoa plantations integrated with native trees ('cabrucas'), edge habitats, and abandoned fields (Raboy et al., 2008).

The illegal capture and trade of wild primates are among the main threats to *C. kuhlii* populations (Oliver & Santos, 1991). Furthermore, there have been reports of the commercialization of *C. kuhlii* on BR-101 in Bahia, facilitating the spread of pathogens to other areas (Neves, 2008). Additionally, *C. kuhlii* is the only arboreal species classified as "Near Threatened" due to traffic accidents in the Atlantic Forest of Southern Bahia (Cassano et al., 2017).

Parasites are important components of ecosystems that regulate host populations. A reduction in weight and fitness is often observed in parasitized animals. A parasite-host balance is usually established, and the host often tolerates helminths. Despite the negative impacts observed on ecosystems from a community perspective, parasites play a fundamental role in the food chain integrity. When an ecosystem is disrupted, parasites are also affected; therefore, monitoring parasites can be used to ensure ecosystem health (Acosta et al., 2020).

Studies addressing the specific interaction of *C. kuhlii* and parasites and linking parasitism to host health parameters are scarce, highlighting the importance of further exploring the topic and reinforcing the need for additional investigations. Therefore, this study aimed to investigate the diversity of helminths in *C. kuhlii* and correlate them with the weights and indices of the livers and gonads of free-living hosts originating from roadkill incidents in Southern Bahia.

## Material and Methods

Thirteen adults of *C. kuhlii* (9 males and 4 females) were recovered after being run over on the Ilhéus-Olivença Highway (BA-001), located at the georeferenced coordinates of approximately 75°15'36"S and 43°37'48"W. The carcasses, collected immediately after being run over and being fresh with a low degree of autolysis, were sent to the Wild Animal Sorting and Rehabilitation Center of the Brazilian company Bahia Mineração (CETRAS/BAMIM) in the city of Ilhéus (Bahia). They were subsequently sent to the Zoology Laboratory of the Multidisciplinary Health Institute, Anísio Teixeira Campus of the Federal University of Bahia (IMS-CAT-UFBA) in Vitória da Conquista (Bahia) for parasitological evaluation. All materials were stored at -20 °C until analysis.

Parasitological necropsies were performed according to the protocol described by Amato & Amato (2010), using an Opton® stereoscopic microscope and surgical instruments (Amato & Amato, 2010). After a detailed viscera examination, the parasites were fixed with a mixture of ethyl alcohol, formaldehyde, and acetic acid (A.F.A.) at 0.85%. After 48 h of fixation, the specimens were transferred to labeled conical microtubes containing 70% alcohol (Amato & Amato, 2010).

Nematodes were cleared in 80% acetic acid, and acanthocephalans were cleared in a beechwood creosote. The digenetic trematodes were subjected to regressive carmine staining (Amato & Amato, 2010). After clearing and staining, helminths were mounted on temporary slides for parasite identification.

Parasite identification was performed based on initial descriptions by Vicente et al. (1997), Machado Filho (1950) and Assis et al. (2021). Morphological and morphometric characteristics were observed, and images were obtained using an Olympus BX-51 microscope with a Q Color 3 camera (Olympus, Tokyo, Japan) and processed using ImagePro Plus software version 4.0. Morphometric data were expressed in millimeters and calculated as the mean ±standard deviation (minimum and maximum values) based on measurements of at least ten specimens (males and females) when possible. Infection descriptors followed the methods described by Bush et al. (1997). To assess the relationship between organ weight (liver and gonads) and helminth distribution, Pearson’s correlation coefficient was used, with a *p*-value set at <0.05 (Barros et al., 2010).

The clinical evaluation of the animals was performed in two stages. First, before the necropsy, the rostro-cloacal length (RCL) was measured using a measuring tape. After determining sex, the body mass (MC) of each specimen was measured using a digital scale and expressed in kilograms. Hepatosomatic (IH) and gonadosomatic (IG) indices were obtained by calculating the ratio between the weight of the analyzed material (WAM; liver for IH and testicles or ovaries for IG) and the weight of the animal (WA) in grams, multiplied by 100 (WAM/WA×100). The results were expressed as percentages (%) (Lanna et al., 2013; Oliveira, 2015).

Principal component analysis (PCA) was performed to describe the relationships between the following variables: RCL, IH, IG, liver weight, presence of parasites in organs (liver, small intestine, and large intestine), and sex of the host (Husson et al., 2017). PCA summarizes the variabilities and relationships of multiple variables in a dataset with two main dimensions (PCA1 and PCA2) explaining most data variation. Therefore, the PCA was used to transform the initial variables into a new set that explains the data variation through two main PCA axes, indicating the relative contribution and correlation of each variable with each PCA dimension (Husson et al., 2017).

## Results

**Figure 1:**
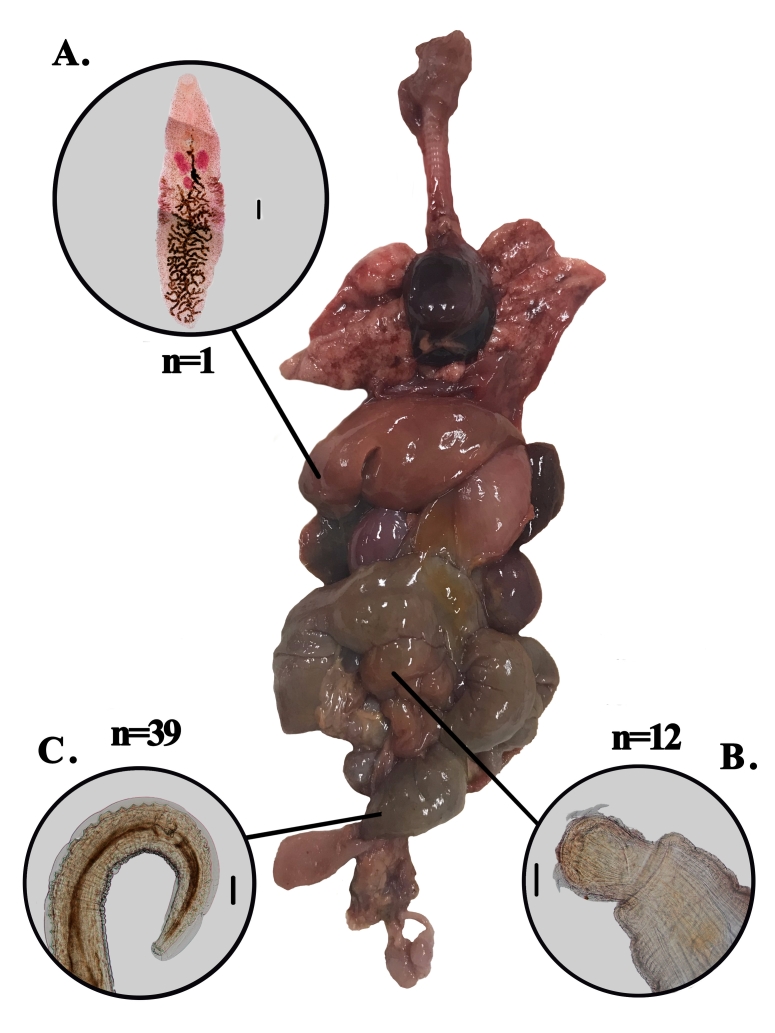
Organs of male *C. kuhlli* with indications of all parasites found in all necropsies, identification of the parasite's location, and 'n' is the total found. A. Representative of *P. illiciens* found in the liver. Scale = 2000μm. B. Representative of *P. confusus* found in the small intestine. Scale = 2000μm. C. Representative of *P. jacchi* found in the large intestine. Scale = 1500μm.

Fifty-one helminths were collected from 30.77% (4/13) of the analyzed *C. kuhlii*. The recovered parasitic species were *Primasubulura jacchi* (7.69%) in male, *Platynosomum illiciens* (7.69%) in male, and *Prosthenorchis confusus* (15.38%) in male and female, which were found in the large intestine, liver, and small intestine, respectively (Figure 1).

The indicators of infection are presented in Table 1. *Primasubulura jacchi* showed the highest mean parasitic intensity and abundance (39 and 3, respectively), followed by *Platynosomum illiciens* (1 and 0.08, respectively) and *Prosthenorchis confusus* (6 and 0.92, respectively).

**Table 1:** Infection indicators observed in helminths recovered from *C. kuhlli* that were victims of roadkill on BR-001, Ilhéus, Bahia.

Helminths	Prevalence	IPM	Intensity Variation	Abundance
NEMATODA				
*Primasubulura jacchi*	7.69%	39	-	3
TREMATODA				
*Platynosomum illiciens*	7.69%	1	-	0.08
ACANTHOCEPHALA				
*Prosthenorchis confusus*	15.38%	6	3 - 9	0.92

IPM - Mean Parasite Intensity.

### *Primasubulurajacchi* (Marcel, 1857) Railliet e Henry, 1913 - Figure 2A, B, C, D, E, F

**Figure 2:**
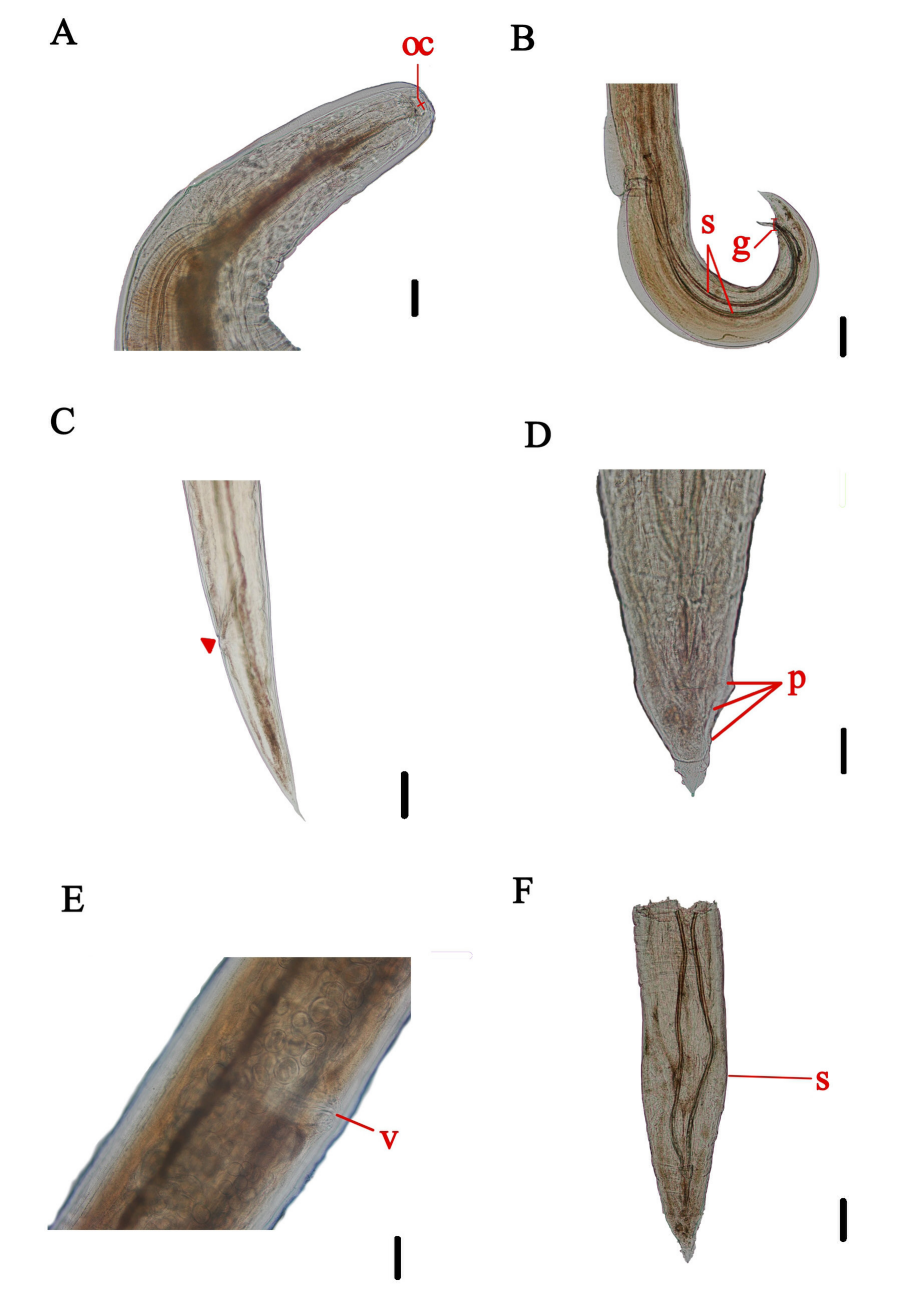
Specimen of *Primasubulura jacchi* recovered from the large intestine of *Callithrix kuhlii* found after roadkill on Highway BR-001, Ilhéus region, Bahia. A. Anterior end of the female, buccal capsule (oc). Scale= 100 μm. Magnification=10x. B. Posterior end of the male, gubernaculum (g), spicules (s). Scale=100μm. Magnification=10x. C. Posterior end of the female indicating the anus. Scale=100 μm. Magnification=10x. D. Posterior end of the male, with some papillae (p) highlighted. Scale= 100 μm. Magnification=10x. E. Vulvar opening (v) of the female. Scale=1500 μm. Magnification= 4x. F. Posterior end of the male, spicule (s). Scale=1500 μm. Magnification= 4x.

General description: whitish nematodes *in vivo*. Thick, transversely striated cuticle. Presence of narrow cephalic wing ending shortly after the end of the esophagus. The anterior end was composed of a cylindrical buccal capsule and an esophagus with a terminal bulbous dilation. The nerve ring was located in the proximal third of the esophagus. The excretory pore opening was posterior to the nerve ring in the middle third of the esophagus. Males with a recurved posterior end, spicules of similar sizes, presence of an elongated gubernaculum, and 11 genital papillae (three pairs pre-cloacal, two pairs cloacal, and six post-cloacal). In females, vulvar openings were located near the middle of the body, and the tail ended at a fine point. The eggs were rounded, thin-shelled, and embryonated near the vulvar entrance.

Habitat: Large intestine

Host: *Callithrix kuhlii*

Morphometric data collected during the study were compared to the original descriptive data of *Primasubulura jacchi* and are presented in Table 2 from males and in Table 3 from females.

**Table 2:** Morphometric characterization of males of *Primasubulura jacchi* (Marcel, 1857) Railet & Henry, 1913. With range of measurements, mean and sample. Measurements in millimeters - Data from the Current Study and Original Morphometric Data.

Features	*Primasubulurajacchi* from current study	*Primasubulurajacchi* from original descriptive data
Total length	11.51±1.72	(8.29 -12.6)
(n=5)	(9.19-14.02)	
Maximum width (esophagus-intestinal junction)	0.46±0.04	(0.50)
(n=5)	(0.40-0.50)	
Buccal capsule with a length	0.03±0.01	(0.033)
(n=5)	(0.03-0.04)	
Buccal capsule with a width	0.03±0.01	(0.033)
(n=5)	(0.03-0.04)	
Esophagus with	1.20±0.07	(1.09)
(n=5)	(1.11-1.28)	
Esophageal bulb with a length	0.29±0.01	(0.26)
(n=5)	(0.28-0.30)	
Esophageal bulb with a width	0.26±0.02	(0.24)
(n=5)	(0.21-0.27)	
Nerve ring from the anterior end	0.30±0.05	(0.30)
(n=5)	(0.27-0.37)	
Excretory pore situated from the anterior end	0.45±0.04	-
(n=5)	(0.38-0.48)	
	
Larger spicule with	1.76±0.08	-
(n=5)	(1.70-1.88)	
Smaller spicule with	1.73±0.07	-
(n=5)	(1.66-1.84)	
Gubernaculum with	0.20±0.03	(0.17)
(n=5)	(0.18-0.25)	
Cloaca situated from the posterior end	0.27±0.02	(0.24)
(n=5)	(0.25-0.30)	

**Table 3:** Morphometric characterization of females of *Primasubulura jacchi* (Marcel, 1857) Railet & Henry, 1913. With range of measurements, mean and sample. Measurements in millimeters - Data from the Current Study and Original Morphometric Data.

Features	*Primasubulura jacchi*	*Primasubulura jacchi*
	from current study	from original descriptive data
Total length	20.78±2.52	(13.9 -19.2)
(n=5)	(17.41-24.41)	
Maximum width (esophagus-intestinal junction)	0.53±0.02	(0.53 -0.66)
(n=5)	(0.51-0.55)	
Buccal capsule with a length	0.04±0.01	(0.033)
(n=5)	(0.03-0.04)	
Buccal capsule with a width	0.04±0.01	(0.053)
(n=5)	(0.03-0.04)	
Esophagus with	1.55±0.10	(1.34)
(n=5)	(1.39-1.66)	
Esophageal bulb with a length	0.33±0.03	(0.34)
(n=5)	(0.29-0.37)	
Esophageal bulb with a width	0.27±0.04	(0.31)
(n=5)	(0.20-0.31)	
Nerve ring from the anterior end	0.52±0.06	(0.32)
(n=5)	(0.43-0.56)	
Excretory pore situated from the anterior end	0.68±0.07	(0.64)
(n=5)	(0.61-0.80)	
Vulval opening located from the anterior end	8.11±1.46	(5.67)
(n=5)	(6.64-10.04)	
Eggs with a length	0.078±0.01	(0.066 - 0.079)
(n=5)	(0.06-0.09)	
Eggs with a width	0.06±0.01	(0.053 - 0.066)
(n=5)	(0.04-0.07)	

### *Platynosomum illiciens* (Braun, 1901) Kossack, 1910- Figure 3

**Figure 3:**
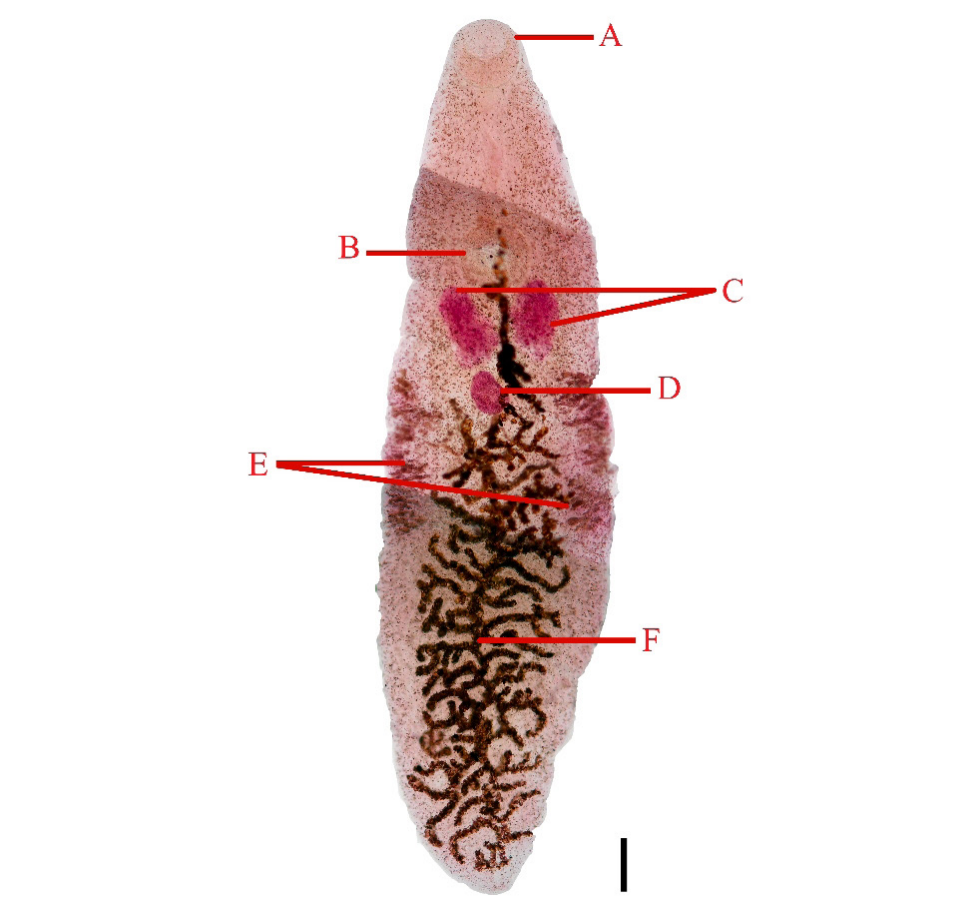
Specimen of *P*. *illiciens* recovered from the liver of *Callithrix kuhlii* individuals found dead on BR-001 highway, Ilhéus, Bahia. A. Oral sucker. B. Ventral sucker. C. Testes. D. Ovary. E. Vitellaria. F. Uterus. Scale=2000 μm. Magnification=20x.

General description of flattened and reddish digeneans *in vivo*. Oral sucker subterminal. Short esophagus and pharynx. The cirrus sac was elongated and pre-acetabular. The circular acetabulum was located in the proximal third of the body. Postacetabular testes were elongated and lobed. Post-testicular ovaries were elongated and lobed. The branched uterus occupied almost the entire body length, filled with thick brown-shelled eggs. The lateral vitellaria were located in the middle third of the body.

Habitat: Liver

Host: *Callithrix kuhlii*

Morphometric data collected during the study were compared to the original descriptive data of *Platynosomum illiciens* and are presented in Table 4.

**Table 4:** Morphometric characterization of *Platynosomum illiciens* (Braun, 1901) Kossack, 1910. With range of measurements, mean and sample. Measurements in millimeters - Data from the Current Study and Original Morphometric Data.

Features	*Platynosomumilliciens* from current study	*Platynosomumilliciens* from original descriptive data
Total length (n=1)	4.68	(5.69-6.38)
Maximum width (widest part of the body) (n=1)	1.23	(1.22-1.53)
Oral sucker with a length (n=1)	0.37	(0.41-0.42)
Oral sucker with a width (n=1)	0.37	(0.38-0,44)
Pharynx with a length (n=1)	0.11	(0.10 -0.11)
Pharynx with a width (n=1)	0.14	(0.11-0.16)
Acetabulum with a length (n=1)	0.41	(0.33-0.44)
Acetabulum with a width (n=1)	0.39	(0.39-0.44)
Left testis with a length (n=1)	0.48	(0.85-0.99)
Left testis with a width (n=1)	0.26	(0.47-0.55)
Right testis with a length (n=1)	0.47	(0.68-1.03)
Right testis with a width (n=1)	0.29	(0.41-0.51)
Ovary with a length (n=1)	0.27	(0.38-0,40)
Ovary with a width (n=1)	0.19	(0.24-0.34)
Left vitellaria with a length (n=1)	0.91	-
Left vitellaria with a width	0.31	-
Right vitellaria with a length (n=1)	0.89	-
Right vitellaria with a width (n=1)	0.29	-
Eggs with a length (n=1)	0.04	-
For Review Only Eggs with a width (n=1)	0.03	-

### *Prosthenorchisconfusus* (Dougherty, 1946) Machado Filho, 1950- Figure 4A, B, C, D, E, F

**Figure 4:**
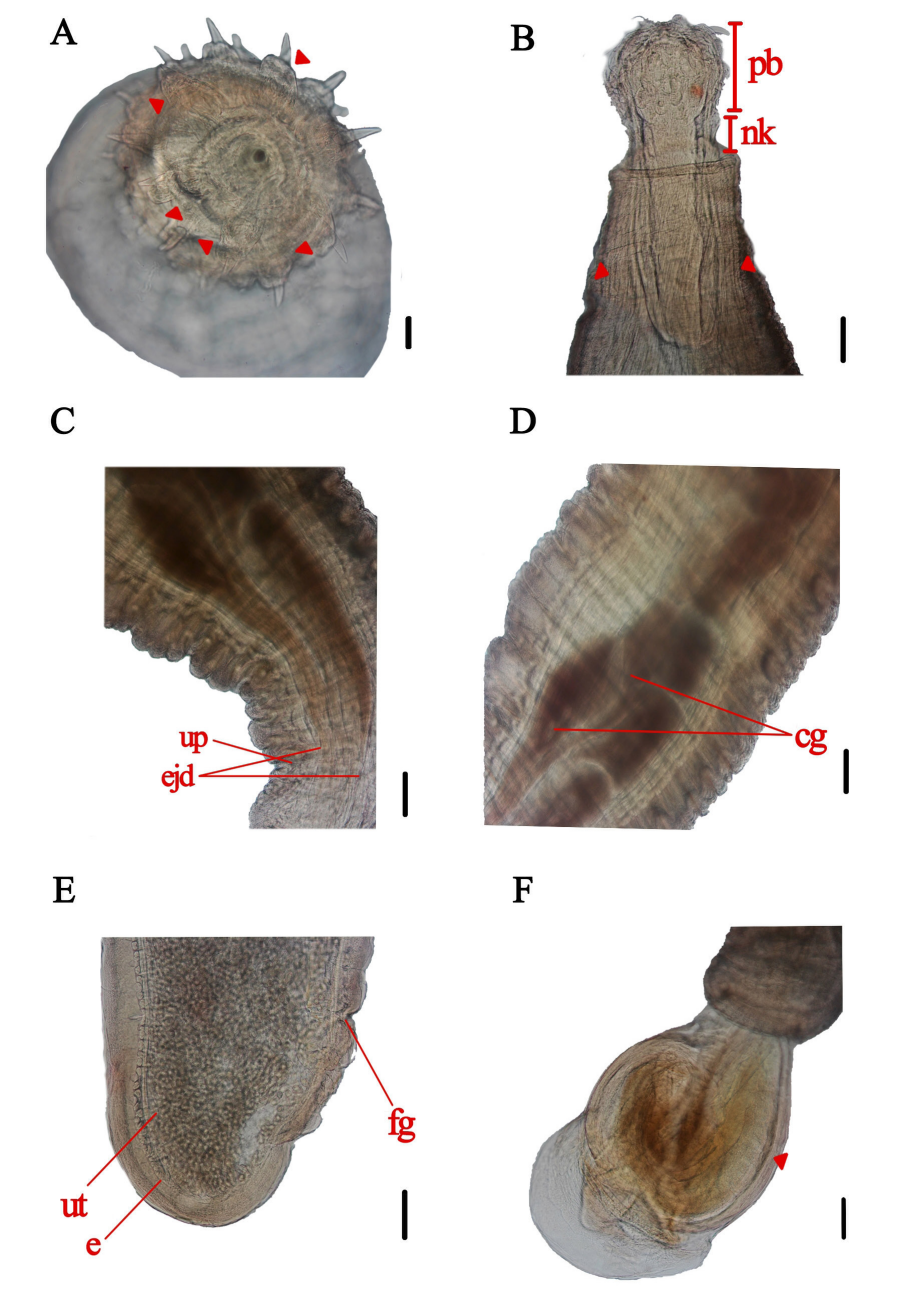
Specimen of *Prosthenorchis confusus* recovered from the small intestine of *Callithrix kuhlii*, found dead on BR-001 highway, Ilhéus, Bahia. A. Cross-section of the proboscis with indication of the 6 hooks of the first row (arrowheads). Scale=100μm. Magnification=10x. B. Anterior extremity composed of the proboscis (pb), neck (nk), and lemnisci (arrowheads). Scale=2000μm. Magnification=20x. C. Posterior extremity of the male, ejaculatory ducts (ejd), urogenital pore (up). Scale=2000μm. Magnification=20x. D. Most posterior region of the male, cement glands (cg). Scale=2000μm. E. Posterior extremity of the female, uterus (ut), eggs (e), female gonopore (fg). Scale=2000μm. Magnification=20x. F. Posterior extremity of the male with protrusion of the copulatory bursa (arrowhead). Scale=2000μm. Magnification=20x.

General description: Acanthocephalans with a cylindrical body, ventrally curved, and whitish *in vivo*. Proboscis globular, armed with six hooks of double and single roots arranged in six rows. Long and flat lemnisci, coiled sometimes. Testes not observed. Four cement glands of distinct sizes, rounded and aligned, followed by wide ejaculatory ducts, were observed. The tail ended in the blunt extremity. Male genital apparatus campanulate, protruding outward in some specimens. The eggs were elliptical with a thick shell and were embryonated when oviposited.

Habitat: Small intestine

Host: *Callithrix kuhlii*

Morphometric data collected during the study were compared to the original descriptive data of *Prosthenorchis confusus* and are presented in Table 5 from males and in Table 6 from females.

**Table 5:** Morphometric characterization of males of *Prosthenorchis confusus* (Dougherty, 1946) Machado Filho. With range of measurements, mean and sample. Measurements in millimeters - Data from the Current Study and Original Morphometric Data.

Features	*Prosthenorchis confuses* from current study	*Prosthenorchis confuses* from original descriptive data
Total length (n=3)	27.78±7.74 (14.54-28.09)	(20-30)
Maximum width (widest part of the body) (n=3)	2.93±0.30 (2.70-3.30)	(1.5-3)
Proboscis length (n=3)	0.63±0.01 (0.62-0.63)	-
Proboscis width (n=3)	0.65 (no variation in measurements)	-
Neck length (n=3)	0.29 (no variation in measurements)	-
Neck width (n=3)	0.53±0.01 (0.52-0.54)	-
Left lemniscus (n=3)	4.91±0	-
Set of cement glands length (n=3)	2.42±0.66 (2.07-3.35)	-
Set of cement glands width (n=3)	0.96±0.40 (0.86-1.59)	-

**Table 6:** Morphometric characterization of females of *Prosthenorchis confusus* (Dougherty, 1946) Machado Filho. With range of measurements, mean and sample. Measurements in millimeters - Data from the Current Study and Original Morphometric Data.

Features	*Prosthenorchis confuses* from current study	*Prosthenorchis confuses* from original descriptive data
Total length (n=2)	29.46±7.03 (24.49-34.43)	(25-30)
Maximum width (widest part of the body) (n=2)	2.75±0.25 (2.57-2.93)	(2-3)
Proboscis length (n=2)	0.67±0.09 (0.60-0.73)	-
Proboscis width (n=2)	0.68±0.028 (0.66-0.70)	-
Neck length (n=2)	0.36 (no variation in measurements)	-
Neck width (n=2)	0.36 (no variation in measurements)	-
Eggs length (n=2)	0.071 ± 0.001 (0.075- 0.072)	0.078
Eggs width (n=2)	0.039 ± 0.002 (0.041 - 0.038)	0.052

**Figure 5:**
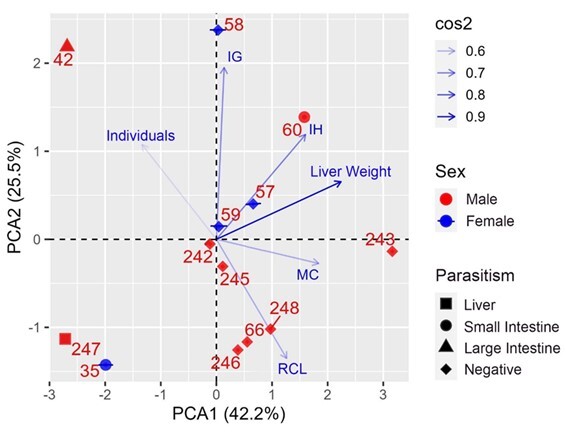
Principal Component Analysis (PCA) to assess relationships among variables related to rostro-cloacal length (RCL), body mass (MC), hepatosomatic indice (IH), gonadosomatic index (IG), and organs where parasites occur (liver, small intestine, large intestine). Cos2 corresponds to the quality of representation of the variables in the principal components according to the Pearson correlation coefficient (p < 0.05). The arrowheads of the different vectors indicate the presence of the highest values of the different analyzed variables, and in the mirrored vectors, they indicate the opposite with the lowest values. The animals' sex (male- ● and female-), absence of infection (Negative), and registration numbers of each animal are indicated.

The two main axes (PCA1 and PCA2) explained the high variability (PCA=67.7%) of all the analyzed data from the RCL, indices, and organs (Figure 5). The first axis (PCA1) accounted for 42.2% of the variation in the data, and liver weight, MC, and IH showed high correlations and relative contributions. Specifically, liver weight exhibited a high, significantly positive correlation with the first axis (R=0.94; p<0.05). However, the liver weight had a low positive correlation with axis 2 (R=0.27; p<0.05). MC (R=0.77, p<0.05), IH (R=0.67, p<0.05), and RCL (R=0.52, p<0.05) also showed a strong positive correlation and explained the high variability in the first axis. Consequently, the second axis (PCA2) accounted for 25.5% of the variability in all data, where IG was the main variable that exhibited a high correlation and contributed to this dimension (R=0.88, p<0.05).

A liver weight, IH, and IG reduction were observed in most infected animals, except for one male callitrichid infected with *P*. *confusus* (60). The latter had the lowest parasitic intensity for helminths, and body mass and IH remained unchanged with parasitism (Figure 5). Infected animals had lower MC and RCL values (42, 247, 35) (Figure 5).

## Discussion

A study on *C. jaccus* in the wild in Rio de Janeiro found at least one parasite in the coprology of 20 individuals that had not been dewormed, representing 50% parasitism in the samples (20/40) (Verona, 2008). In the first study conducted with *C. kuhlii*, two carcasses were collected in the Una Biological Reserve (15°09'S, 39°10'W) in southern Bahia. The necropsy analyses showed the presence of six helminths in each individual (100% parasitism, 2/2) (Catenacci et al., 2016). Tavela et al. (2013) demonstrated that of free-living Callitrichidae in areas with a high level of human contact, 86% of the fecal samples of individuals were parasitized (44/51). In another study analyzing the viable feces of *Callithrix* spp., 50% of the individuals were infected by helminth (17/34) (Santos Sales et al., 2010). In the present study, the percentage of parasitized marmosets was 30.77%, a low value compared to other studies on hosts of the same species and genus (<50%).

*Primasubulurajacchi* is a nematode present in primates, specifically in *Saguinus* spp., *Callicebus* spp., and *Callithrix* spp. (Sarmiento et al., 1999; Michaud et al., 2003; Pacheco et al., 2003; Melo, 2004; Tavela et al., 2013). There have been reports in Brazil, mainly of *Callithrix* spp. (Melo & Pereira, 1986; Santos Sales et al., 2010). *P. jacchi* infection was the most frequent, with the highest parasitic intensities (39) and average abundance (3) among all recovered helminths. The identification of *P. jacchi* was based on morphological characteristics, primarily whitish coloration, thick cuticle, presence of a narrow cephalic wing extending beyond the end of the esophagus, distinctive curvature at the posterior end, and spicules of similar sizes in males. Additionally, an elongated gubernaculum and 11 genital papillae (three pairs pre-cloacal, two pairs cloacal, and six post-cloacal) were noted. In contrast, in females, analysis of morphological characteristics was also crucial for identification. The vulvar opening was positioned near the midpoint of the body while the tail was tapered (Railliet & Henry, 1913).

*Platynosomumilliciens* is a digenetic trematode that primarily parasitizes the biliary tract, particularly in felids such as domestic cats. However, its occurrence has also been reported in birds and other mammals, including rodents and non-human primates such as *Callithrix penicillata* (Pinto et al., 2017). Specific identification was primarily based on the analysis of morphological and morphometric characteristics, as well as the arrangement of the ovaries, testes, and uteri of the recovered helminths. These trematodes had a reddish coloration and a flattened body. A notable feature was the presence of an oral sucker located immediately below the anterior end of the body. Furthermore, the esophagus and pharynx are short. More specific details revealed an elongated cirrus pouch located before the acetabulum, which, in turn, is circular and located on the proximal part of the parasite's body. The arrangement of the reproductive organs is also a distinctive feature. The testes, positioned after the acetabulum, are elongated and lobed. The ovary, located after the testes, exhibited an elongated and lobulated shape. Remarkably, the branched uterus occupies most of the body length. The uterus was filled with thick, brown-shelled eggs. In addition, lateral vitellaria were observed in the middle third of the body. This combination of distinct morphological characteristics is crucial for identifying *P. illiciens* and contributes to a more comprehensive understanding of its taxonomy and biology.

The genus *Prostenorchis* was originally described by Travassos (1915a), with *Prostenorchis elegans* as the type species. The spelling of its generic name was revised by Travassos (1915b). Stiles & Hassall (1920) suggested that the type species is *Echinorhynchus elegans* (Diesing, 1851) from *Cebus sciureus*, rather than *Prosthenorchis elegans* (Olfers, 1816) from *Mergus merganser*. Machado Filho (1950) reviewed the specimens collected by Travassos and asserted that they were *Prosthenorchis sigmoides*, as previously described by Meyer (1932). Therefore, he adopted *P. sigmoides* as the type species. Machado Filho (1950) described 13 new species from various hosts, including primates, bats, and carnivores. Petrochenko (1958) rejected all the new species described by Machado & Castro (2019), accepted *Prosthenorchis spirula* (Olfers, 1819) as the type species, and included *Prosthenorchis elegans* (Diesing, 1851); *Prosthenorchis novellae* (Parona, 1890); *Prosthenorchis curvatus* (v. Linstow, 1897); *Prosthenorchis avicula* (Travassos, 1916); *Prosthenorchis lühei* (Travassos, 1916), and *Prosthenorchis sigmoides* (Meyer, 1933) within the genus.

This is the first record of *P. confusus* in the marmoset *C. kuhlii*. The last time *P. confusus* was described in the intestines of *Cebus* spp. from Pacáu, Minas Gerais, Brazil (Machado Filho, 1950). The genus *Prostenorchis* has previously been observed in *Callithrix* spp., possibly because of the distribution of *C. kuhlii* marmosets in edge habitats and fragments (Tavela et al., 2013). This species was identified based on the quantity and arrangement of hooks in the proboscis. Although most of the morphometric characteristics of the recovered specimens resemble those described by Travassos (1915a), it is still differentiated by the author as *Prosthenorchis spirula* (Olfers, 1819) due to differences in the number of hook series, dimensions, and characteristics of male and female genital organs. It is characterized by a slightly developed proboscis armed with six series of strong hooks, not five, each with six hooks; the first three series have double roots, and the arrangement of the hooks is oblique. Additionally, it has a rough body and a thick neck. The proboscis merged with the body without a differentiated neck region. Wide and bell-shaped male copulatory pouch (Figure 4F). These characteristics are similar to those described by Machado Filho (1950). This is the first record of the host *C. kuhlii*, and its location is the same as that of the small intestine, as in the previous identification.

In marmosets, *P. elegans* infection is transmitted through cockroaches, with perforations in the intestine. Nineteen of the 20 *Oedipomidas oedipus* that died were infected with *P. elegans*, and two had intestinal perforations with peritonitis. The intestinal wall has deep crater-shaped cavities with necrosis, inflammation, and granulation tissues (Verona, 2008). In the present study, it was observed that the presence of parasites in the liver affected its weight, and the presence of parasites in the large and small intestines, regardless of the sex of the host. Most uninfected individuals showed higher values of MC and RCL when compared to the infected individuals. Thus, the presence of helminths may indicate compromised organs and development in the studied callitrichids.

Host tolerance/resistance to the parasite depends on the incidence and severity of parasitism, with parasitic intensity being an important factor in understanding the health of these animals (Ewald, 1983). The low parasitic intensity observed in one of the animals in this study appeared to be related to the milder deleterious effects of helminths, as IH (hepatosomatic indice) and body mass values did not significantly change with parasitism. In contrast, individuals with higher parasitic intensities showed lower IH, IG (gonadal indice), body mass, and RCL values, suggesting a negative influence of higher parasitic loads on the general conditions of the hosts (Figure 5).

As the habitats where marmosets live and develop are being destroyed or fragmented, there is an increasing proximity between them and humans, which can allow for the exchange of pathogens between these two species and an increased risk of disease transmission (Michaud et al., 2003; Carvalho-Filho et al., 2006). Additionally, it is crucial to understand that among the endemic mammals in the region are the Wied's marmoset (*C. kuhlii*) and the golden-headed lion tamarin (*Leonthopithecus chrysomelias*) (Kinzey, 1982). *C. kuhlii* shares areas with *L. chrysomelas*, raising the potential for parasite transmission between them, which could compromise conservation efforts for critically endangered golden-headed lion tamarin (Tavela et al., 2013).

## Conclusions

This is the first study on *C. kuhlii* in the wild and roadkill specimens. The helminths identified in *C. kuhlii* included *P. jacchi*, *P. illiciens*, and *P. confusus*. This study reported the infection of *Callithrix* spp. by *P. confusus* for the first time. The occurrence of *P. jacchi* and *P. illiciens* in the Callitrichidae was confirmed. PCA revealed a general pattern of organ weight reduction (liver and gonads) in the presence of parasites. Furthermore, higher MC and RCL values were associated with negative parasitic records. The present study is crucial to expand our knowledge of the health of this vulnerable species and provide valuable information to assist in conservation efforts for *C. kuhlii* preservation.
